# Effective thresholds for reporting suspicions and improve early detection of avian influenza outbreaks in layer chickens

**DOI:** 10.1038/s41598-018-26954-9

**Published:** 2018-06-04

**Authors:** Jose L. Gonzales, Armin R. W. Elbers

**Affiliations:** Department of Bacteriology and Epidemiology, Wageningen Bioveterinary Research, P.O. Box 65, 8200 AB Lelystad, The Netherlands

## Abstract

The objective of this study was to identify effective reporting thresholds for suspicions of both highly pathogenic (HPAI) and low pathogenic avian influenza (LPAI) outbreaks in layer farms. Daily mortality and egg-production data from 30 Dutch farms with no record of AI infection were analysed and thresholds set. Mortality rates above or egg-production below these thresholds for two consecutive days would trigger an alarm sign. The following thresholds were identified for mortality: (i) A mortality threshold of 0.08% or 0.13% for layers kept indoors or with free-range access respectively, (ii) a 2.9 times higher mortality than the average weekly mortality of the previous week, and iii) a moving-average threshold that could be implemented for each specific farm. For egg-production: (i) a weekly ratio lower than 0.94 in egg-production drop, and (ii) a moving-average threshold. The accuracy of these thresholds was assessed by quantifying their sensitivity, specificity and time to trigger disease detection using data from 15 infected and 31 non-infected farms. New thresholds were more sensitive and signalled infection two to six days earlier than the presently used thresholds. A high Specificity (97–100%) was obtained by combining mortality and egg production thresholds in a serial approach to trigger an alarm.

## Introduction

Early detection of outbreaks of infectious diseases in poultry largely depends on the rapid identification and reporting of clinical signs by the farmer or animal health professionals to the competent authorities. Production parameters such as daily mortality, egg production and feed and water intake are routinely monitored in many commercial poultry farms worldwide. Sudden changes in these parameters could be indicators of outbreaks of infectious diseases. For example, a sudden increase in mortality for consecutive days^1^ has been associated with outbreaks of highly pathogenic avian influenza (HPAI) and exponentially increasing mortality is frequently used as reference for reporting suspicions and aid early detections of this disease^2^.

In the Netherlands, different reporting thresholds have been used as references for farmers and veterinary practitioners to notify suspicions of HPAI. First, a daily mortality higher than 0.5% in a poultry flock was implemented as a reporting threshold in the framework of precautionary measures (high alert phase) in the Netherlands at the time that a HPAI epidemic in Italy was evolving in 1999–2000^3^. Later, during the HPAI H7N7 epidemic in 2003, an additional weekly mortality threshold >3% was established as trigger for reporting of a suspect situation to the competent authorities^4^. Once the epidemic was controlled, analysis of flock mortality data resulted in the recommendation to establish an updated reporting threshold ≥0.25% mortality/day for two consecutive days in layer flocks^4^. This recommendation was, however, not implemented exactly as proposed, but in an adjusted form in article 84 of the Statutory Regulation “Prevention, eradication and monitoring of contagious animal diseases, zoonosis and TSE (2005)”^5^: the reporting threshold was set at ≥0.5% mortality/day for two consecutive days in layer flocks. In addition, the following provision was implemented in the law: a poultry farmer has to consult his veterinary practitioner if in AI-susceptible animals the following conditions occur: a reduction in feed or water intake of ≥5% per day seen for two consecutive days or a drop in egg production of ≥5% observed for two consecutive days in layers or reproduction animals. The first new HPAI-epidemic in commercial poultry in the Netherlands since 2003 occurred in 2014, when a H5N8-subtype was introduced in the country, likely by migrating wild birds^6^. A total of five commercial poultry flocks became infected^6^ and a preliminary evaluation of the course of clinical events in the outbreak flocks indicated that (i) it took several days after the start of increased mortality to reach the official reporting threshold; (ii) the reporting threshold was often interpreted by poultry farmers at the farm level instead of at the poultry house level (in which the clinically diseased chickens were present), also resulting in delayed reporting. It has been estimated that the application of the current mortality threshold results in a mean herd incubation time – from time of infection till detection - of 12 (range 11–15) days for HPAI outbreaks^7^. Use of a more effective threshold could lead to earlier reporting and subsequent detection of a HPAI-infection which is crucial to decrease the infectivity of the infected flock and with that to prevent spread of the virus to other flocks^8^.

The reporting threshold in the Statutory Regulation was originally intended for detection of HPAI outbreaks. However, HPAI outbreaks can evolve from low pathogenic avian influenza (LPAI) infections^9^, which based on empirical evidence, are not easily nor rapidly detected by using this threshold. As a follow-up development to the Dutch reporting threshold, Beltran-Alcrudo *et al*.^10^ for LPAI virus introductions and Malladi *et al*.^11^ for HPAI virus introductions have explored mortality threshold signals that abandoned fixed alert levels, and brought it to a next level by using expected flock-tailored mortality and moving-average mortality. The objective of our study was to make a next step and identify effective reporting thresholds for suspicions of both HPAI and LPAI outbreaks based on both increased mortality and drops in egg production in commercial layer farms.

## Material and Methods

### Data

#### Healthy flocks

Retrospective daily mortality, egg production and feed and water intake records were provided by the poultry industry from 61 apparently healthy commercial layer flocks which either were kept indoors (barn system) or with free-range access (here referred to as outdoors). These records were from flocks seronegative for avian influenza during their production cycle, therefore day one of our analysis begins when pullets were placed in the barns to start production around 17–18 weeks of age. No molting was done in any of the flocks analysed and the median (range) production cycle of these flocks was 406 (366–483) days. Data came in the form of production calendars manually recorded, therefore calendars from each flocks were transferred to Excel spread sheets. Once data was digitalized it was clear that for many flocks there was too much information missing about feed and water intake, hence these parameters were not included for analysis.

To assess the expected “baseline” daily mortality and egg production and to determine potential thresholds for reporting, 30 complete production calendars were used, which were provided first for this study. Six out of these 30 were production calendars from outdoor flocks and 24 production calendars from indoor flocks. We will refer to these calendars as the “development dataset”.

The remaining 31 production calendars (15 from outdoor and 16 from indoor flocks) were used to assess the specificity of the developed reporting algorithms. This subset will be referred to as the “validation dataset”.

#### Infected flocks

Retrospective mortality and production records were obtained from confirmed outbreaks with either HPAI or LPAI virus. These records came from 7 flocks affected with the H5N8 HPAI virus either in 2014 (n = 4)^6^ or 2016 (n = 3)^12^, and 8 records from LPAI detected outbreaks (2013–2017). Additionally, mortality data from 110 infected flocks (86 commercial layers and 24 layer breeders) with the H7N7 HPAI virus during the epidemic in 2003 were obtained^4^.

### Detection algorithms

#### Daily mortality

Three types of reporting thresholds based on daily mortality were assessed: (i) mortality threshold, (ii) a mortality ratio thresholds and (iii) weekly moving average threshold.

i) Mortality thresholds.- These are fixed parameters which work as a kind of “population” trigger. All records from the development dataset were used to assess the expected “baseline” daily mortality in Dutch layer flocks. Daily mortality was assessed by fitting generalised linear models (GLM) or mixed models (GLMM) where a Poisson, Negative binomial and Gaussian distributions were assessed. A negative binomial model had the best fit and therefore was the selected model. In this model the number of dead chickens was modelled as a function of days in production (this included, introduction of spline terms) and production type (indoor, outdoor). Daily population of live chickens in the flock was included as an offset and the flock identification as random variable. The daily mean mortality and corresponding 97.5 quantiles were calculated. Mortality thresholds were then set based on these 97.5 quantiles.
ii) Mortality ratio.- This threshold has the advantage that it will adjust to a flock particular production characteristics since it uses the flocks recent mortality history to inform the level of mortality expected at the time of surveillance, therefore detecting mortality changes from the recent past. This ratio is calculated daily during the whole production cycle as follow (1):
  1 $${R}_{ij}=\frac{{d}_{ij}}{{M}_{j-1}}$$
  where *R*_*ij*_ is the mortality ratio of day *i* (Monday = 1, ….., Sunday = 7) in week *j* (production week 1, 2, .. 60), *d*_*ij*_ is the number of chickens that died at day *i* in week *j* and ${M}_{j-1}$ is the average mortality the previous week (sum of daily mortality/7) (See supplementary information, Tables S1 and S2, for an example). Although this ratio adjust to the particular flock characteristics, a fixed threshold value had to be set as reference (population level) for reporting. To this end, a similar approach to that taken to calculate the mortality threshold was taken. GLMM were fitted where Poisson, Negative binomial and Gaussian distributions were assessed. The Negative binomial model showed the best fit. For this model, daily mortality *d*_*ij*_ was used as the response variable, ${M}_{j-1}$ was introduced as an offset, day in production (with one spline term) and production type as explanatory variables and the flock identification as random variable. The “population” mean mortality ratio and 97.5% quantile were calculated. These 97.5% quantile were used to set the threshold ratio for triggering an alarm.
iii) CUSUM mortality.- This is a seven-day moving average method which is a flock tailored trigger. Similar to the mortality ratio, the CUSUM-based method uses recently observed data (previous days) in a particular flock to inform the expected mortality in this specific flock at the time of surveillance. We used the Early Aberration Reporting System (EARS) algorithm developed by^13^. Two algorithms were assessed, the first one “EARS-1” triggers an alarm (detection) when the current mortality at day *d*_*i*_ is greater than the baseline mean plus three standard deviations (sd). The baseline mean and sd are based on the mortality of the previous 7 days. The second algorithm “EARS-2” differs from the EARS-1 in the introduction of a two days lag between the baseline mean and the day ${d}_{i+1}$ of evaluation.

#### Daily egg production

Similar to using daily mortality, three types of reporting thresholds were assessed which were based on the detection of drops in egg production: (i) a fixed threshold, (ii) egg production ratio and iii) CUSUM method.

i) Fixed thresholds.- different production indicators were assessed as fixed triggers. We moddelled for example the expected production rate as a function of days in production and estimated the 2.5% quantile of the (lowest) expected daily production, we also explored a parameter we called daily variation in production, which was the difference between the production fraction at ${d}_{i-1}$ and current production fraction. Variation in daily egg production was large, hence none of these indicators worked reliably. These indicator signalled many false alarms per production cycle (data not shown).
ii) Egg production ratio.- The method used was the same as that for the mortality ratio, with the difference that for this ratio the production fraction (total egg/chicken population) was used for the calculations. A GLMM with a Poisson distribution had the best fit during the analysis. Using this model the “population” mean egg production ratio and 2.5 and 0.5% quantiles were calculated for the production period. These quantiles were used to set the threshold ratio for triggering an alarm.
iii) CUSUM-egg.- The EARS-1 and EARS-2 algorithms were assessed to target drops in egg production, which was expresed as fraction (egg production/chicken population). Different from the CUSUM-mortality, the CUSUM-egg algorithm triggers an alarm (detection) when the current egg production at day *d*_*i*_ is lower than the baseline mean plus two sd. Three sd were initially tested but appeared to be less sensitive (data not shown).

Data analysis was done using the statistical package R^14^, GLMM were fitted using the library lme4^15^. The R codes for the EARS algorithms were those made available by Watkins *et al*.^16^.

### Evaluation of performance

Performance assessment was based on three parameters: (1) sensitivity (Se), which describes the ability of the reporting threshold to raise a “true” alarm any time during an outbreak period of HPAI or LPAI in layers; timeliness, which describes how early an alarm was triggered in relation to the time of detection of an outbreak; and specificity (Sp), which describes the ability of the reporting thresholds to not raise an alarm (“false alarms”) during the whole production cycle of not infected flocks.

A positive alarm was defined as any signal (mortality or egg production) occurring for two consecutive days during an outbreak period or the production cycle (two LPAI outbreaks were detected by serological monitoring).

To assess Se and timeliness, records from infected flocks were used. To assess timeliness, which is important as an early detection indicator, the time (day) an alarm is detected by the proposed thresholds was compared with the actual time of detection of the outbreaks. To assess Sp, records from the validation subset were used. In addition to classifying an alarm as false, the expected number of false alarms (false alarm rate) triggered by a threshold per day in a population of commercial layer farms was quantified.

In addition to the newly developed thresholds in this study, the performance of the reporting threshold of mortality ≥0.5% (acting Statutory Regulation^5^) and ≥0.25%^4^ per day for two consecutive days was also assessed. Performance was also assessed when these thresholds were passed for one day because during a HPAI epidemic, poultry farms with suspicious clinical signs are culled rapidly.

### Data availability

The data that support the findings of this study are available from several different individual poultry farmers and the Netherlands’ Food and Consumer Product Safety Authority but restrictions apply to the availability of these data, which were used under license for the current study, and so are not publicly available. Data are however available from the authors upon reasonable request and with permission of several different individual poultry farmers and the Netherlands’ Food and Consumer Product Safety Authority.

## Results

### Development of thresholds

**Figure 1:**
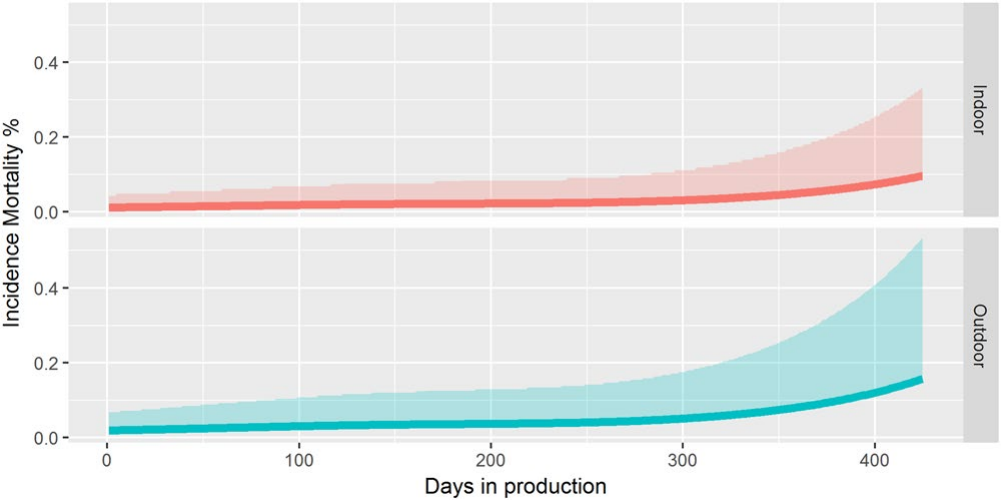
Expected daily mortality and corresponding 97.5% quantiles in indoor (layers kept inside the barn, for the whole production cycle) and outdoor (layer have free range access) layer flocks in the Netherlands.
ii. Mortality and egg production ratios.- No differences between production systems were observed when assessing the ratios estimated for each flock. Variation in mortality due to age is also reduced since ratio values are updated based on the mortality of the recent past (previous week), and values are centred around 1. The mean ratio and 97.5% quantiles (mortality) or 0.5% quantiles (egg production) were quantified using a negative binomial or Poisson model respectively (best fits). A threshold ratio for mortality was set to >2.9, which was the median of the estimated 97.5% quantiles for the production cycle. The threshold ratio for egg production was set to <0.94 (0.5% quantile) or <0.96 (2.5% quantile).
iii. CUSUM-methods.- Initial assessments showed that the EARS-2 algorithm, which uses a two days lag, performed better (higher Se and Sp) than the EARS-1 (data not shown). Therefore the EARS-2 algorithm was used for both mortality and egg production. In Fig. 2, it is given a graphical example of these CUSUM algorithms as well as the other detection thresholds.

i. Fixed thresholds.- We assessed fixed thresholds for mortality and egg production. As explained in the methods section, initial inspection did not show reliable results for the use of a fixed threshold for egg production (many false alarms per production cycle), hence no such thresholds were set for this parameter. However, it was possible to set thresholds for mortality. Baseline mortality rates in layer farms were best described by a GLM with a Negative binomial distribution. Daily mortality was on average 1.6 (95% confidence intervals: 1.5–1.8) times higher (p < 0.001) in outdoor layer farms (organic and not organic) than in layer farms which kept layers indoors during the whole production cycle. In Fig. 1 the estimated mean daily mortality and corresponding 97.5% quantiles are shown. An increase in daily mortality is expected when layers get older, with a marked increase after one year in production. Therefore, based on the estimated quantiles, we subjectively set two thresholds. The first threshold was the median of the estimated quantiles during the production cycle (highest production cycle in our data 483 days) and the second was the third quantile. These fixed thresholds (>360 days in production) were: >0.08% (0.14%) daily mortality for indoor layer farms and >0.12% (0.21%) daily mortality for outdoor layers. Higher thresholds were also assessed for flocks older than 360 days, however these higher thresholds had very poor sensitivity (data not shown).

**Figure 2:**
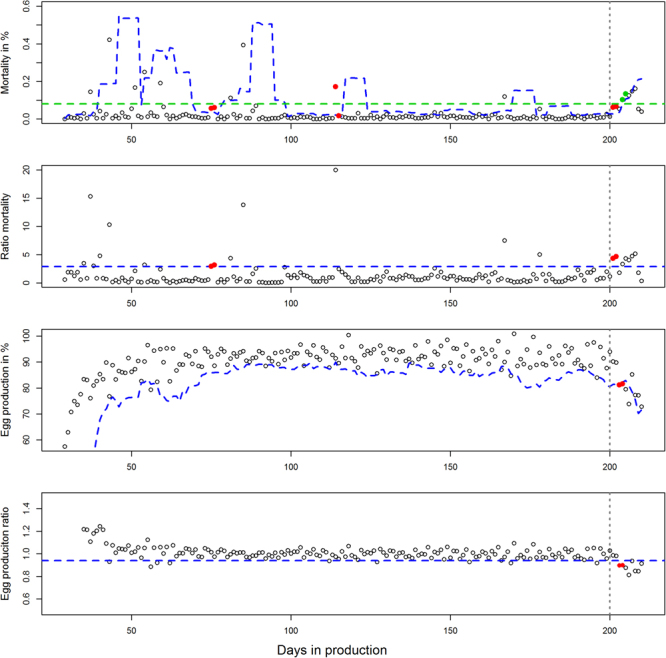
Application of the different reporting thresholds developed for detection of suspicions of avian influenza outbreaks in layers. The figure shows an example of these thresholds applied for the detection of a low pathogenic outbreak in a commercial layer flock with an indoor (barn) production system. From up to bottom, the first graph shows both the CUSUM-mortality (blue dotted lines) thresholds and the fixed threshold of 0.08% mortality (green dotted line). The second graph shows the ratio-mortality threshold >2.9 (orange dotted line), the third graph shows the CUSUM-egg production thresholds and the fourth (bottom) graph shows the ratio-egg production <0.94 threshold. Points coloured in red or green (first graph marking detection using the fixed mortality threshold) are detected alarms. True alarms are those detected later than day 200 (vertical dotted line) of production.

### Performance of detection thresholds

#### Sensitivity (Se)

We first assessed sensitivity using mortality data from the H7N7 HPAI epidemic in 2003. These data had information of daily mortality for the last seven days before detection and culling, therefore we could only assess the fixed mortality threshold and the mortality ratio. To assess the mortality ratio, a mean mortality (from the 110 infected flocks (see material and methods)) at day 1 (day −7) was calculated and used as the denominator (${M}_{j-1}$). Our mortality rate (0.08%) and the ratio thresholds correctly triggered an alarm in 95.3% and 97.3% of the affected farms, respectively. The Se was significantly higher than that using a mortality threshold of >0.25% or 0.5% (Table 1).

**Table 1:** Sensitivity (with 95% lower and upper confidence limits) of different mortality thresholds to signal flocks infected with H7N7 highly pathogenic avian influenza.

Mortality trigger	Sensitivity % (detected/total)	95% LCL – UCL
Mortality % >0.08/0.13	95.3% (105/110)	89.8–98.0
Ratio >2.9	97.3% (107/110)	92.3–99.1
Mortality % ≥0.25% (1 day)	73.6% (81/110)	64.7–81.0
Mortality % ≥0.5% (1 day)	73.6% (81/110)	64.7–81.0

Data from 110 infected layer flocks during the 2003 epidemic in the Netherlands was used for this assessment.

We then assessed the Se using production records (data available from start of production to detection of outbreak) from more recent outbreaks (2013–2017). Mortality thresholds were assessed with LPAI subtype H5/H7 (n = 9) and HPAI (n = 7) outbreaks. Egg production thresholds were only assessed using records from 6 LPAI and 6 HPAI outbreaks. No production records were available from the other outbreaks (Table 2).

**Table 2:** Ability and timeliness of mortality and egg production thresholds to signal highly and low pathogenic avian influenza outbreaks.

	HPAI		LPAI	
	No. detected/No. tested (%)	Days earlier detection (range)	No. detected/No. tested (%)	Days earlier detection (range)
Mortality				
CUSUM	5/7 (71.4)	2 (0–6)	8/9 (89.0)	6 (0–89)
Ratio >2.9	5/7 (71.4)	2 (0–6)	7/9 (77.8)	7 (3–89)
Mortality % >0.08/0.13	5/7 (71.4)	2 (0–5)	6/9 (66.7)	5 (3–89)
Mortality % >0.25% (2 day)	3/7 (43.0)	0 (0–1)	0/9 (0.0)	
Mortality % >0.5% (2 day)	3/7 (43.0)	0 (0–1)	0/9 (0.0)	
Mortality % >0.25% (1 day)	6/7 (85.7)	0 (0–2)	0/9 (0.0)	
Mortality % >0.5% (1 day)	5/7 (71.4)	0 (0–2)	0/9 (0.0)	
Egg production				
CUSUM	1/6 (16.7)	0	3/6 (50.0)	6 (2–6)
Ratio <0.94	2/6 (33.3)	0 (0–1)	4/6 (66.7)	6 (1–7)

Data from recent outbreaks (2012–2016) were used for this analysis.

For HPAI detection, the newly developed thresholds triggered an alarm in 5 (71%) out of the 7 HPAI outbreaks and detection was on average two days earlier than the actual detection time, whilst the fixed 0.25% and 0.5% thresholds only detected 3 outbreaks if used as recommended or 6 or 5 outbreaks respectively if the alarm is triggered the day the threshold is passed. Egg production thresholds were not sensitive to detect the assessed outbreaks, the ratio threshold performed the best, but it only triggered an alarm in 2 (33%) out of the 6 outbreaks with no gain in timeliness (Table 2).

As for LPAI detection, all three newly developed mortality thresholds triggered an alarm in most of the outbreaks (Table 2), with the CUSUM and ratio thresholds appearing to be the most sensitive. These last two thresholds detected the outbreaks on a median time of 6 to 7 days earlier than the actual detection day. The >0.25% and >0.5% mortality thresholds did not detect (triggered an alarm) any of the outbreaks. The egg production thresholds appear to be more sensitive for detection of LPAI than HPAI outbreaks. The ratio <0.94 appears to be the most sensitive threshold. Detection time of egg production thresholds was around one day later than detection based on mortality (Table 2).

#### Specificity (Sp)

Table 3 shows the estimated Sp for the existing thresholds (mortality >0.5% or >0.25%) and each of the newly developed thresholds. The existing thresholds showed the highest Sp (100% and 90.3% respectively). The CUSUM (mortality and egg production) and the ratio-egg production thresholds had poor Sp (<40%), with these thresholds on average 2.25 to 3.23 false alarms would be triggered per day in a population of 1000 layer farms. Better Sp were observed for the mortality >0.08/0.13% (87.1%) and ratio mortality (71%) thresholds. To improve Sp we assessed the use of the newly developed thresholds in a serial approach, where one threshold is used as screening (CUSUM- or ratio-mortality) and if the threshold is positive a second threshold (mortality >0.08/0.13%, CUSUM- or ratio-egg production) is used for confirmation. By using a serial approach specificities, for any serial combination, were higher than 90% and the expected false-alarm rates (range 0.00 to 0.15) per day per 1000 farms were lower than using only one threshold (Table 3). The serial approach however delays triggering of an alarm by one to two days.

**Table 3:** Specificity of reporting thresholds when used alone or in a serial approach.

Thresholds	Specificity %	False alarm (FA) rate. Number FA/day X 1000 farms
Mortality		
Mortality % ≥0.5 (1 day)^a^	100.0	0.00 (0.00–0.27)
Mortality % ≥0.25 (1 day)^a^	90.3	0.22 (0.05–0.65)
Mortality % ≥0.25 (2 day)^b^	100.0	0.00 (0.00–0.27)
Ratio >2.9^b^	71.0	0.79 (0.33–1.26)
CUSUM^b^	35.5	2.25 (1.53–2.97)
Mortality % >0.08/0.13^b^	87.1	0.39 (0.02–0.77)
Egg production		
Ratio production <0.94^b^	38.7	2.37 (1.59–3.15)
CUSUM^b^	22.7	3.23 (2.25–4.22)
Serial use of thresholds^c^		
Ratio-mortality		
Mortality % >0.08/0.13	97.0	0.07 (0.00–0.41)
CUSUM- egg	100.0	0.00 (0.00–0.27)
Ratio production	97.0	0.07 (0.00–0.41)
CUSUM-mortality		
Mortality % >0.08/0.13	93.5	0.15 (0.02–0.53)
CUSUM-egg	93.5	0.15 (0.02–0.53)
Ratio production	96.8	0.07 (0.00–0.41)

^a^An alarm in triggered the first day the threshold is passed.

^b^For these thresholds an alarm is triggered when the threshold is passed for at least two consecutive days.

^c^A screening threshold (Ratio-mortality or CUSUM-mortality) triggers an alarm when a threshold is passed for at least two consecutive days. The confirmation threshold needs to be passed for only one day following the alarm triggered by the screening.

## Discussion

The objective of this study was to identify effective reporting thresholds for early detection of AI outbreaks in Dutch layer flocks. Reporting thresholds based on daily mortality and egg production data were identified, which showed high Se but low Sp, the latter being clearly observed for egg production thresholds. This limitation in Sp is dealt with when mortality and egg production thresholds are used in a serial approach (e.g. mortality as screening and egg production as confirmatory test), resulting therefore in triggering suspicions of both HPAI and LPAI infections with high accuracy.

The mortality thresholds presented in this study are sensitive for detection of suspicions of both HPAI and LPAI outbreaks. As for detection of HPAI, they are more sensitive than the previously suggested thresholds of 0.25%^4^ and therefore also for the 0.5% threshold set in the acting Statutory Regulation. The latter has not been effective for the detection of the last HPAI outbreaks in the Netherlands and given that it was not implemented for detection of suspicions of LPAI, it did not detect any of the LPAI outbreaks. The latter confirms the low Se previously shown for detection of LPAI outbreaks^10^. The 0.25% or the 0.5% thresholds were identified using the data from the HPAI H7N7 epidemic in the Netherlands in 2003, whilst the newly developed thresholds are updated to the current production systems in the country, with layer flocks no longer kept in cages but in large groups in barns either indoors or with free-range access. This change in production system may influence the transmission dynamics in a flock leading to different mortality rates, which no longer are timely detected by the “old” thresholds.

The production system also influences the expected baseline mortality for a “healthy” flock, with higher mortality observed in outdoor (free-range) farms than farms keeping layers indoors. Additionally, age of the layers influences mortality, with a significant increase in mortality at the end of the production cycle. Hence, different fixed mortality thresholds had to be set for each production type and production period (younger or older than 365 days in production). The CUSUM and mortality-ratio thresholds are not affected by the production system or age of the layers. These thresholds are tailored to the flock and adjust the baseline in time, making them (the methodology) easier to extrapolate to other production systems (both thresholds) including other poultry species (only CUSUM). However, we still assessed a fixed mortality threshold because it is easier to explain to - and to be implemented by - the farmers, who are the end-users of these thresholds. In this scenario the mortality-ratio can also be easily implemented by the farmer since many of them, as seen in the Netherlands, are still recording daily production parameters on paper forms (Figure S1). The CUSUM algorithm will require that data is recorded electronically and farmers have an inbuilt app, spreadsheet or computer programme doing the analysis.

We assessed timelines, by retrospectively looking at the difference in days between the actual detection time of an outbreak and the time (in days) the alarms would have been triggered if our thresholds were in place as part of the early warning system (EWS). Most HPAI outbreaks analysed were detected by passive surveillance (following confirmation of the first outbreak), when awareness is high and surveillance is intensified^2^. Therefore Se and timeliness are likely underestimated, and the fact that the novel thresholds appear to reduce time of detection on average by 2 days in situations under intensified surveillance indicates that such thresholds could be also used during emergency surveillance. However the Sp of these thresholds under emergency conditions – with farmers reporting any perceived increase in mortality - needs to be assessed. As for LPAI, these thresholds detected the outbreaks between 5 and 7 days earlier. Furthermore, these thresholds will improve Se of the surveillance system as a whole since they detected outbreaks that were not reported (due to subtle changes in mortality) and were later detected (n = 2) by serological surveillance.

Se using egg production thresholds appear to be lower than using mortality thresholds and in case of LPAI, detection (raised alarm) was usually a day later than the detection time using mortality. As for HPAI, egg-production thresholds appear to have a low Se. These thresholds only detected the index cases (n = 2), and the alarms (pass threshold for two consecutive days) were triggered the same day as actual detection. This drop in egg-production happening later than the increase in mortality were also observed for the LPAI outbreaks analysed by Beltran-Alcrudo *et al*.^10^.

For these thresholds to be effective, they need to balance the potential benefit of the early detection of an outbreak (following a true alarm) with the cost of responding and attending the false alarms they trigger. Clinical signs in layer chickens after infection with AIV are not pathognomonic, with sudden increases in mortality and/or drops in egg production being caused by a range of other non-notifiable infectious diseases or management related problems (e.g. diet related problems, etc), which likely triggered alarms in the assessed “healthy flocks” in our study. These non-AI related changes in mortality and egg-production affect the Sp of the thresholds here evaluated, with the CUSUM-mortality and the egg-production thresholds showing low Sp, when used alone, which can lead to trigger on average 2–3 false alarms per day in a population of 1000 layer farms. A way to circumvent this low Sp is to combine thresholds in a serial approach. We show that combining mortality thresholds (CUSUM or Ratio) with the fixed mortality >0.08/0.13% threshold (which works better for HPAI) or the egg-production thresholds considerably improves Sp, with the expected false alarm rates dropping to between 0.07 to 0.15 false alarms per day in a population of 1000 layer farms. This translates into around 24 to 49 false alarms per year for the Netherlands (n = 899 layer farms), which is similar to the current number of false alarms reported yearly in the country (Dr. Bruschke, Ministry of Agriculture, Nature Management and Food Quality, personal communication).

The use of a serial approach however could compromise Se and timeliness, this is particularly the case when using mortality with egg production thresholds for the reporting of HPAI outbreaks. Therefore, we assessed the serial approach by triggering the alarm when the confirmatory threshold is passed for 1 day instead of two. This improved Sp, kept similar level of Se and affected timeliness only by one day. Given the exponential increase in mortality caused by HPAI infections^4^, the best performing approach in such scenario would be to use either the ratio or CUSUM mortality as screening and the incidence mortality as confirmatory threshold.

A limitation of our study lays within the quality of record keeping by the farmers. A very common observation was the inaccuracy from record keeping of mortality and egg production data, with the latter observed with higher frequency. This resulted in low counts of egg on one or two days followed by high counts the following day or days. This happened frequently during weekends, which triggered alarms compromising in some cases the specificity of the developed thresholds. Additionally, the quality of records for food and water intake was poor for most of the farms, which made it impossible to use these parameters for the development of detection thresholds. Recording data electronically and raising awareness of farmers about the benefits of accurately recording production data would improve performance of any reporting algorithm. Additionally, we only had data from a few outbreaks to assess Se. With more data we could further extend the analysis and be able the compare thresholds with higher accuracy and select the best performing ones. As for the Sp, the study used retrospective data from “healthy flocks”, and it was not possible to follow up and confirm the causes of the false alarms (regarding AI). The fixed and ratio thresholds here developed represent baseline limits for layer farms and they could also be assessed for reporting of other diseases.

We used a regression model to model the baseline mortality and define fixed thresholds, these models could also be used in a similar approach as Beltran-Alcrudo *et al*.^10^. However, this approach would require the availability of a software or spreadsheet to implement the model predictions and compare daily mortality. Given this requirement, we opted, instead, to use the ratio or the CUSUM approach. The former is a new approach, it can be easily implemented and it is robust as it adapts to the individual characteristics of a flock. It is however necessary to set a baseline threshold by assessing this ratio in a population of healthy flocks. CUSUM has been previously shown to be sensitive for detection (trigger alarms) of HPAI^11^. Initial assessment indicated that the EARS-1 algorithm, which is similar to the method reported by Malladi *et al*.^11^, appeared to be less sensitive than the EARS-2, and therefore we proceeded only to fully assess the EARS-2 algorithm. This apparent higher Se of the EARS-2 is in agreement with an assessment made of the EARS algorithms with simulated data^17^.

Whilst thresholds set in this study are specific for the Dutch layer industry, the methods can be easily extrapolated to other poultry production sectors (eg. broilers, ducks, turkeys, etc) in the Netherlands or other countries. The European Union’s diagnostic manual for avian influenza^18^ has stablished procedures to be performed when attending suspected outbreaks. Clinical thresholds are exclusively set when attending suspicions (in contact holdings and holdings within the protection and surveillance zones) following confirmation of a HPAI outbreak. Two of the set thresholds are: daily increase in mortality >3 times the normal mortality rate and depression (>5%) in daily egg-production. The mortality threshold is similar to the ratio-mortality threshold suggested in this study and we provide evidence that this is a sensitive threshold (for chicken layers) which can also be used in the absence of HPAI confirmed outbreaks to improve early warning systems^2^. As for egg-production, we show that its drop is neither a sensitive nor timely indicator of HPAI suspicions, it is however a useful threshold for LPAI suspicions^18^. Furthermore, no clinical thresholds are set for LPAI in the diagnostic manual and here we provide evidence that reference thresholds can be also set for LPAI suspicions.

To conclude, a series of methods were assessed to identify effective thresholds for reporting suspicions of AI outbreaks in commercial layer flocks. These thresholds are based on assessment of daily mortality and egg production, which need to be combined in a serial approach to optimise their efficacy. It is clear that the existing threshold (mortality ≥0.5%), needs to be updated and we provide new effective thresholds. Additionally, we tried other two approaches and we found that a new threshold, the ratio-threshold, which requires only one week of information, is an effective and simple approach to be implemented either manually or electronically. The later would be ideal and could help farmers manage flock health more efficiently.

## Electronic supplementary material

Supplementary Information
