# Supplementary material for: Effective thresholds for reporting suspicions and improve early detection of avian influenza outbreaks in layer chickens
Source: Sci Rep. 2018 Jun 4;8:8533. doi: 10.1038/s41598-018-26954-9 (PMC5986775; doi:10.1038/s41598-018-26954-9)
Supplement: Supplementary file 1 — Supplementary Information [file 41598_2018_26954_MOESM1_ESM.pdf]

Effective thresholds for reporting suspicions and improve early detection of avian influenza outbreaks in layer chickens

J.L. Gonzales and A.R.W. Elbers

S1. Quantification and application of weekly ratio

This is an example using data of one of the outbreaks analysed in this study. The figure bellow (Figure S1) is an example of the way daily production is manually recorded in a Dutch layer farm.

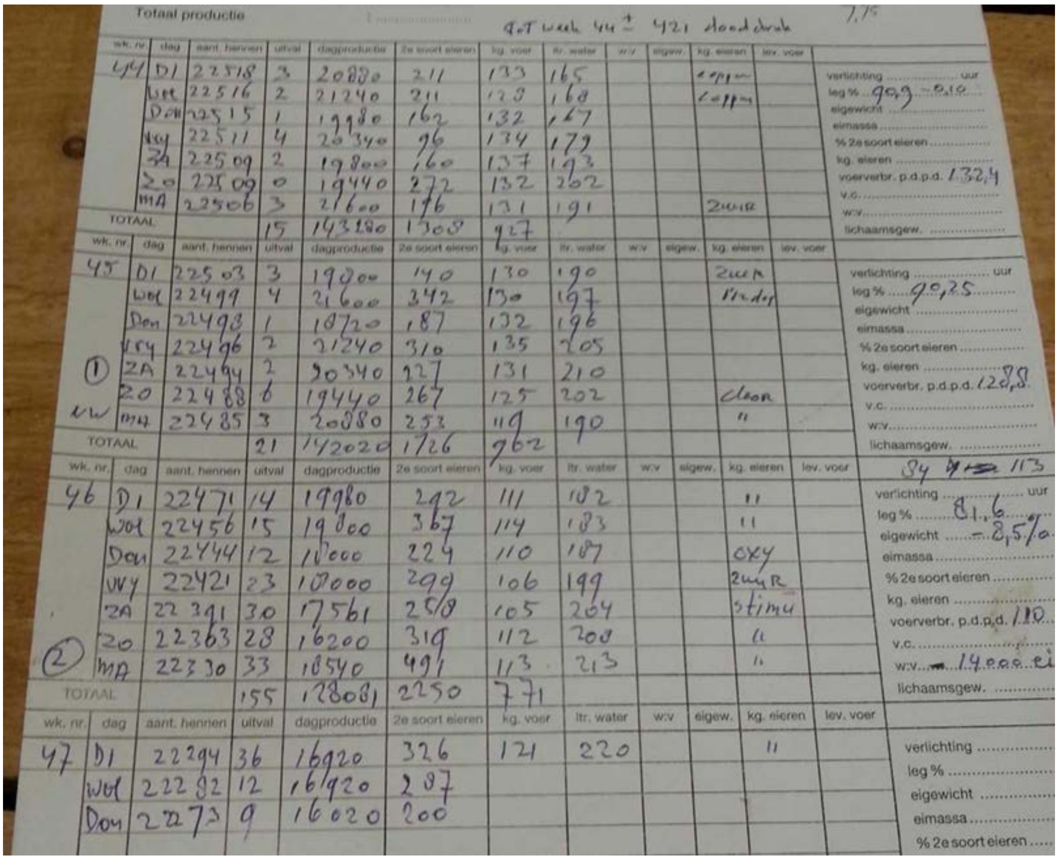

Totaal productie

wk. nr.	dag	aant. hennen	uitval	dagproductie	2e soort eieren	kg. voer	ltr. water	w:v	eigew.	kg. eieren	lev. voer
44	DI	22518	3	20880	211	133	165				
	Wol	22516	2	21240	211	128	168				
	Don	22515	1	19980	162	132	167				
	Vry	22511	4	20340	96	134	179				
	ZA	22509	2	19800	160	137	193				
	ZO	22509	0	19440	272	132	202				
	MA	22506	3	21600	176	131	191				
TOTAAL			15	143280	1308	927					
45	DI	22503	3	19800	140	130	190				
	Wol	22499	4	21600	342	130	197				
	Don	22498	1	18720	187	132	196				
	Vry	22496	2	21240	310	135	205				
	ZA	22494	2	20340	127	131	210				
	ZO	22488	6	19440	267	125	202			clean	
	MA	22485	3	20880	253	119	190			"	
TOTAAL			21	142020	1726	962					
46	DI	22471	14	19980	242	111	182			"	
	Wol	22456	15	19800	367	114	183			"	
	Don	22444	12	18000	224	110	187			oxy	
	Vry	22421	23	18000	299	106	199			24uR	
	ZA	22391	30	17561	258	105	204			stimu	
	ZO	22363	28	16200	319	112	208			"	
	MA	22330	33	18540	491	113	213			"	
TOTAAL			155	128081	2250	771					
47	DI	22294	36	16920	326	121	220			"	
	Wol	22282	12	16920	287						
	Don	22273	9	16020	200						

Figure S1. Picture of production logbook in Dutch layer farm.

The total number of chickens dead in week 44 were 15 (see figure, row “Totaal”, column 4). The average mortality per day for week 44: 15/7 = 2.1. this value is used to calculate the daily mortality ratio for week 45 as shown in Table S1

Table S1. Calculation of daily mortality ratio for week 45

Week 45	Day	Mortality	Ratio	
	Tue	3	3 / 2.1 =	1.4
	Wed	4	4 / 2.1 =	1.9
	Thu	1	1 / 2.1 =	0.5
	Fri	2	2 / 2.1 =	1.0
	Sat	2	2 / 2.1 =	1.0
	Sun	6	6 / 2.1 =	2.9
	Mon	3	3 / 2.1 =	1.4

The average mortality per day for week 45: 21/7 = 3. This value is then used to calculate the daily mortality ratio for week 46 as shown in table S2.

Table S2. Calculation of the daily mortality ratio for week 46.

Week 46	Day	Mortality	Ratio	
	Tue	14	14 / 3 =	4.7
	Wed	15	15 / 3 =	5.0
	Thu	12	12 / 3 =	4.0
	Fri	23	23 / 3 =	7.7
	Sat	30	30 / 3 =	10.0
	Sun	28	28 / 3 =	9.3
	Mon	33	33 / 3 =	11.0

This week the outbreak is detected on Tuesday since the mortality ratio > 2.9 for two consecutive days.
